# Long-Term Treatment with n-3 Polyunsaturated Fatty Acids as a Monotherapy in Children with Nonalcoholic Fatty Liver Disease

**DOI:** 10.4274/jcrpe.1749

**Published:** 2015-06-03

**Authors:** Mehmet Boyraz, Özgür Pirgon, Bumin Dündar, Ferhat Çekmez, Nihal Hatipoğlu

**Affiliations:** 1 Turgut Özal University Faculty of Medicine, Department of Pediatric Endocrinology and Diabetes, Ankara, Turkey; 2 Süleyman Demirel University Faculty of Medicine, Department of Pediatric Endocrinology and Diabetes, Isparta, Turkey; 3 Katip Çelebi University Faculty of Medicine, Department of Pediatric Endocrinology and Diabetes, Izmir, Turkey; 4 Gülhane Military Medical Academy, Department of Pediatrics, Ankara, Turkey; 5 Erciyes University Faculty of Medicine, Department of Pediatric Endocrinology and Diabetes, Kayseri, Turkey

**Keywords:** Nonalcoholic fatty liver disease, n-3 polyunsaturated fatty acids, adolescent, obesity, insulin sensitivity

## Abstract

**Objective:**

To investigate the efficacy and safety of n-3 polyunsaturated fatty acids (PUFA) treatment in obese children with nonalcoholic fatty liver disease (NAFLD).

**Methods:**

One hundred and eight obese (body mass index (BMI) >95th percentile for age and sex) adolescents with NAFLD were included in the study. Mean age of the subjects was 13.8±3.9 years (9-17 yrs). The diagnosis of NAFLD was based on the presence of liver steatosis with high transaminases. The subjects were randomly divided into two groups. Group 1 (PUFA group, n=52) received a 1000 mg dose of PUFA once daily for 12 months and lifestyle intervention. Group 2 (placebo group, n=56) received a recommended diet plus placebo and lifestyle intervention for 12 months. Insulin resistance was evaluated by homeostasis model assessment of insulin resistance (HOMA-IR) from fasting samples.

**Results:**

BMI, fasting insulin levels and HOMA-IR values in both groups decreased significantly at the end of the study. In group 1, 67.8% of the patients had a decrease from baseline in the prevalence of steatosis (p<0.001). Frequency of elevated alanine aminotransferase (ALT) levels (39.2% to 14.2%; p<0.01) and elevated aspartate aminotransferase (AST) levels (25% to 17.8%; p=0.01) decreased significantly in the PUFA group. Following a 12-month diet plus placebo and lifestyle intervention treatment, 40.3% (21) of the patients in the placebo group also showed a decrease in frequency of steatosis (p=0.04) and slight decreases in frequency of elevated ALT levels (38.4% to 28.8%; p=0.01) and AST levels (30.7% to 28.8%; p>0.05).

**Conclusion:**

Our results indicated that n-3 PUFA treatment is safe and efficacious in obese children with NAFLD and can improve ultrasonographic findings and the elevated transaminase levels.

## INTRODUCTION

Nonalcoholic fatty liver disease (NAFLD) is one of the most prevalent causes of chronic liver disease in children, with rates ranging from 10% to 77% based on ethnicity, diagnostic criteria and associated morbidities, including obesity and insulin resistance (1,2). Owing to the increasing prevalence of NAFLD and the potential for nonalcoholic steatohepatitis (NASH) to progress to cirrhosis and liver-related mortality, more research has focused on therapy of this important liver disease over the last two decades. These studies have included lifestyle modification, use of pharmacological agents and surgical intervention. Non-pharmacological measures are aimed at reducing caloric intake and increasing physical activity levels. Weight loss and increased physical activity are effective in NAFLD treatment. Modest weight loss (7% to 10%) and exercise improve liver histology, insulin sensitivity and quality of life and should form the backbone of any treatment strategy (3,4,5).

Various potential therapies that are thought to address the underlying pathogenetic mechanisms of pediatric NAFLD have been extensively investigated in the past two decades, including the role of lipid-lowering agents, insulin sensitizers, antioxidants and cytoprotective agents. In small pilot studies, treatment with the insulin sensitizer, metformin and the antioxidant vitamin E were reported to improve liver enzyme levels but not to reduce histological damage in children with NAFLD (3,4,5,6,7).

Omega-3 polyunsaturated fatty acids (PUFA), especially eicosapentaenoic acid (C20: 5n3, EPA) and docosahexaenoic acid (C22: 6n3, DHA), by regulating gene transcription factors (i.e., PPARα, PPARγ, SREBP-1, ChREBP), can control key pathways involved in hepatic lipid metabolism (8,9). In more detail, PUFA are potent activators of PPARα, which up-regulates several genes involved in the stimulation of fatty acid oxidation (10,11,12,13) and down-regulates pro-inflammatory genes, such as TNF-α and IL-6 (14). Moreover, PUFA activate PPARγ resulting in increased fat oxidation and improved insulin sensitivity (15). It has been shown that PUFA treatment is effective in NAFLD. A diet enriched in PUFA was shown to improve insulin sensitivity and reduce intrahepatic triglycerides content and steatohepatitis, in both mice (16,17,18,19) and rats (7,20) with fatty liver.

In the present double-blind 12-month study, we aimed to investigate the effect of PUFA vs. placebo on liver functions, liver brightness and insulin resistance in obese adolescents with NAFLD undergoing a nutritional programme of a balanced calorie diet plus daily physical exercise.

## METHODS

### 

One hundred and thirty-eight obese patients with NAFLD (65 girls and 73 boys, aged 9 to 17 years, mean age: 13.9±3.7 years) attending the outpatient clinic of the İstanbul Şişli Etfal Education and Research Hospital from March 2010 to June 2012 were initially selected for the study.

The subjects were accepted as obese when the calculated body mass index (BMI) was above the 95th percentile for age and sex (according to the charts developed by the National Center for Health Statistics and the National Center for Chronic Disease Prevention and Health Promotion, US, 2000). BMI was calculated as the weight in kilograms divided by the square of the height in meters. To compare BMI across different ages, BMI z-scores were also calculated. The z-score represents the number of standard deviation (SD) above or below the considered population mean value based on standardized tables for children (21). Obesity was defined as a BMI z-score value above 2 SD for age and gender.

In addition to obesity, inclusion criteria were persistently elevated serum aminotransferase levels, diffusely echogenic liver in imaging studies suggestive of fatty liver, exclusion of hepatic virus infections, no alcohol consumption, no history of parenteral nutrition and no use of drugs known to induce steatosis (e.g. valproate, amiodarone or prednisone). All obese adolescents with abnormally high transaminases and abnormal liver ultrasound were screened for other liver conditions (hepatitis B surface antigen, hepatitis C antibody, prothrombin time, iron, total iron-binding capacity, ferritin and antinuclear antibodies) that were all negative.

Of the 138 patients, only 108 (53 girls and 55 boys) completed the protocol and were included in the analysis. The mean age of this group was 13.7±3.6 years (range 9 to 17 yrs) and mean BMI SD score (BMI-SDS) was 2.7±0.4. The study was conducted in accordance with the guidelines proposed in the Declaration of Helsinki and was approved by the Ethics Committee of İstanbul Şişli Etfal Education and Research Hospital. Written informed consent was obtained from all participants and in case of minors, from their parents.

The same pediatric endocrinologist assessed the pubertal development stage in all subjects, using the Tanner criteria. Sexual maturation stage was >2 in all patients (Tanner stages II-V).

Blood pressure was measured using a mercury sphygmomanometer with an appropriate cuff size after a minimum ten-minute rest. We used the National High Blood Pressure Education Program Working Group (2004) normal values for children as a reference to evaluate blood pressure measurements (22). A blood pressure measurement ≥95th percentile for age, sex and height was considered as hypertension.

The patients were randomly assigned to a 12-month double-blind treatment. They were divided randomly into two groups and the ultrasonography operator was blinded to the groups. Group 1 (PUFA group) included 56 obese children who received an adequate diet plus a 1000 mg dose of PUFA (MarincapR Special 1000 mg; Kocak-Farma Company) once daily for 12 months and also lifestyle intervention. Group 2 (placebo) included 52 obese children, who received a diet plus placebo and lifestyle intervention. Parents were asked to personally verify and record the daily intake of the medications. Compliance was tested by pill counting and amount of weight loss. Compliance was considered to be good when patients took more than 90% of the pills provided, as verified by counting residual pills at the next visit to the outpatient department. During the 12-month treatment period, liver enzymes and fasting lipids were monitored at months 3, 6, 9 and 12 of the follow-up. Hepatic fat infiltration was detected by upper abdominal ultrasonography at month 12.

### Diet and Lifestyle Intervention

The recommended diet was composed of 50% carbohydrates, 20% protein and 30% fat, in accordance with the American Heart Association diet. All obese patients were advised to lose weight with a restriction of daily caloric intake to 25-30 kcal/kg per day (23). The lifestyle intervention programme consisted of scheduled exercise (three times per week for 1 hour) and the promotion of self-initiated physical activities.

### Blood Samples and Insulin Sensitivity Markers

Laboratory tests were performed at baseline and every 3 months. All measurements, including the fasting blood glucose, insulin and plasma transaminases (ALT and AST), were done on blood samples obtained in the morning after an overnight fast. Lipid profiles [triglycerides, total cholesterol, high-density lipoprotein cholesterol (HDL-C) and very-low-density lipoprotein cholesterol (VLDL-C)] were also obtained from determinations performed on fasting blood samples. Low-density lipoprotein cholesterol (LDL-C) value was calculated using Friedewald equation. Serum lipid profiles were measured using a modular analytical system (Roche/Hitachi). The glucose oxidase method was applied in the determination of blood glucose levels. Insulin levels were measured using a radioimmunoassay kit (Diagnostic Products, Los Angeles, CA, USA). Insulin resistance was analyzed using the homeostasis model assessment of insulin resistance (HOMA-IR). HOMA-IR was calculated applying the following formula: [fasting insulin (mIU/L) x fasting glucose (mmol/L)/22.5]. A HOMA-IR value greater than 3.16 was used to determine insulin resistance in pubertal patients (24,25). Hypertransaminasemia (or elevation of serum ALT) was defined as serum ALT levels exceeding the upper normal limit at least 1.5 times (5).

### Liver Ultrasonography

Blinded ultrasonographic evaluation of liver brightness was assessed by the same physician at baseline and after the 12-month treatment. Liver ultrasound examination was performed by an experienced radiologist, using a high-resolution B-mode ultrasound system (Logic 400; GE, Milwaukee, WI, USA) having an electric linear transducer midfrequency of 3-5 MHz. The radiologist was blinded to all clinical and biochemical characteristics of the subjects. NAFLD, if present, was classified based on the severity of fatty liver based on standard criteria (26,27) and graded as below:

### Grade 1 (mild):

A slight diffuse increase in fine echoes in the hepatic parenchyma with normal visualization of the diaphragm and intrahepatic vessel borders.

### Grade 2 (moderate):

A moderate diffuse increase in fine echoes with slightly impaired visualization of the diaphragm and intrahepatic vessels.

### Grade 3 (advanced):

A marked increase in fine echoes with poor or no visualization of the intrahepatic vessel borders, diaphragm and posterior portion of the right lobe of the liver.

### Statistical Analysis

The data were presented as mean ± SD and analyzed using SPSS 20 for Windows (SPSS, Chicago, IL, USA). Statistical analysis for baseline characteristics of the study groups was performed using χ2 test and t-test. Student’s t-test and Wilcoxon signed rank test were applied to evaluate the changes in biochemical parameters before and after treatment. P<0.05 was considered statistically significant.

## RESULTS

### 

Baseline clinical and demographic data on the treatment and placebo groups are shown in Table 1. There were no differences between the two groups by gender, age and height. There were also no significant differences between the two groups in terms of biochemical findings and frequency of hepatic steatosis at baseline. The ultrasound stages of steatosis were paired (p=0.63). No patient was classified as grade 0. In group 1 (PUFA group), 37% of the children were classified as grade 1, 48% as grade 2 and 15% as grade 3. In group 2 (placebo), 30% of the patients were classified as grade 1, 56% as grade 2 and 14% as grade 3.

### Post-Treatment Data

After 12 months of diet plus PUFA therapy and lifestyle intervention, BMI significantly decreased from 29.7±4.8 to 23.7±3.5 (p=0.01) in the PUFA-treated group. This group had significantly higher HDL-C (45.4±6.9 vs. 38±8.6 mg/dL, p=0.02) and lower triglyceride levels (53.3±22.3 vs. 65.8±23.3 mg/dL, p=0.01) than the placebo group at the end of the study. The placebo group also showed a significant reduction in BMI (from 27.2±3.3 to 23.6±2.56, p=0.03) at the end of the 12 months. The systolic and diastolic blood pressures remained unchanged over 12 months in this group.

In the PUFA group, fasting insulin concentrations declined from 13.4±8.7 IU/L to 7.4±4.9 IU/L (p=0.008), while these values remained unchanged in the placebo group (11.5±9.9 to 9±4.4 IU/L, p>0.05). In addition, fasting glucose values were significantly lower after 12 months (86±18.4 vs. 72.4±8.9 mg/dL, p=0.001) in the PUFA group as compared to group 2. Insulin sensitivity also improved significantly with PUFA treatment and HOMA-IR significantly decreased (2.98±1.45 vs. 1.9±1.2, p=0.006) (Table 2).

As shown in Table 2, changes in BMI at the end of the study were similar in the two groups (p>0.05). However, when comparing the changes of HOMA-IR between the groups, the subjects receiving a diet plus n-3 PUFA treatment and lifestyle intervention (group 1) showed significantly improved metabolic control for fasting insulin levels (p=0.04) after 12 months. The PUFA group also showed significant changes in lipid levels after 12 months compared to the placebo group. Accordingly, except for fasting total cholesterol and LDL-C values, the PUFA group, showed a significant improvement in triglycerides and HDL-C levels as compared to the placebo group (p<0.05). There were no significant changes in diastolic blood pressures among the groups. However, systolic pressure decreased in the PUFA-treated group (125±7.5 vs. 103±15 mmHg, p=0.01) (Table 2).

**Figure 1:**
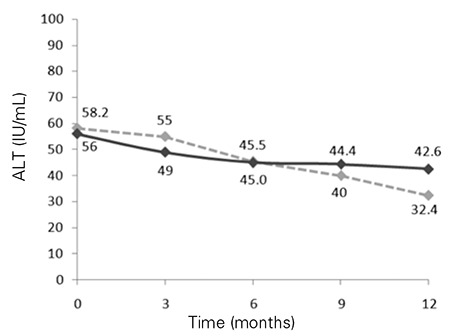
Mean values for alanine aminotransferase (ALT) levels during follow-up in the two groups [black line for polyunsaturated fatty acids (PUFA) group].

**Figure 2:**
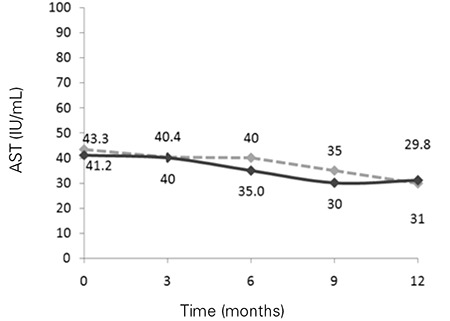
Mean values for aspartate aminotransferase (AST) levels during follow-up in the two groups [black line for polyunsaturated fatty acids (PUFA) group].

As compared to the pre-treatment values, ALT and AST levels at 3, 6, 9 and 12 months decreased significantly in the two groups over the study period (p<0.01) (Figures 1, 2). Both groups showed significant reductions in ALT and AST levels, but these improvements were more pronounced in the PUFA group compared to the placebo group.

**Figure 3:**
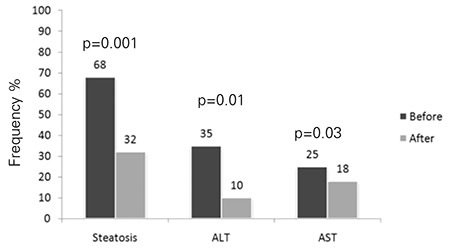
Frequency of steatosis, elevated alanine aminotransferase (ALT) and elevated aspartate aminotransferase (AST) before (black bar) and after (grey bar) the 12-month treatment in the polyunsaturated fatty acids (PUFA) group.

**Figure 4:**
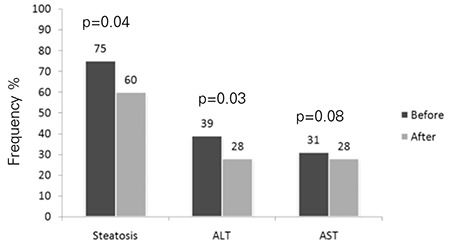
Frequency of steatosis, elevated alanine aminotransferase (ALT) and elevated aspartate aminotransferase (AST) before (black bar) and after (grey bar) the 12-month treatment in the placebo group.

In the placebo group, 40.3% (n=21) of the patients showed a decrease from baseline in steatosis (p=0.04). The frequency of elevated serum ALT decreased from 38.4% (n=20) to 28.8% (n=15) and that of elevated serum AST from 30.7% (n=16) to 28.8% (n=15) (p=0.03; p>0.05, respectively). In the PUFA group, on the other hand, 67.8% (n=38) of patients had a decrease from baseline in the frequency of steatosis (p=0.001) and the frequency of elevated serum ALT fell from 39.2% (n=22) to 14.2% (n=8) and that of elevated serum AST from 25% (n=14) to 17.8% (n=10) (p=0.01; p=0.03, respectively) (Figures 3, 4).

## DISCUSSION

### 

In this study, we evaluated the efficacy and safety of PUFA in the treatment of obese adolescents with NAFLD. The results showed that, compared to a treatment schedule consisting of a diet and lifestyle intervention, at the end of a treatment period of twelve months, PUFA significantly improved the HOMA-IR values, systolic blood pressure, ALT, AST, fasting insulin and triglycerides levels and also normalized the ultrasonographic findings. In 67.8% of patients in the PUFA group, ultrasonography showed a reduction in fatty liver disease (p<0.001). However, an improvement in fatty liver findings were also found in 40.3% of patients in the placebo group. Tanaka et al (28) reported that treatment with EPA, one of the major components of n-3 PUFA, seems to be safe and efficacious for patients with NASH. In our study also, no severe side effects were observed during the treatment period.

Increased fat intake with an excessive amount of n-6 fatty acids can promote NAFLD (29,30). Two pilot clinical trials support the protective role of n-3 PUFA in NAFLD. Capanni et al (31) evaluated the efficacy of prolonged n-3 PUFA supplementation in 56 patients with an ultrasonographic diagnosis of NAFLD. 1000 mg/day of n-3 PUFA was administered to 42 subjects for 12 months. At the end of the treatment, subjects showed a significant improvement of NAFLD compared with controls. In addition, n-3 PUFA supplementation was associated with a significant reduction in liver enzymes, fasting glucose and triglyceride levels. The second study was a non-controlled trial in 23 NASH patients who were supplemented with 2700 mg/day of EPA for one year. Seven of the 23 patients underwent post-treatment liver biopsy which showed improvement of hepatic steatosis and fibrosis, hepatocyte ballooning and lobular inflammation in six patients (28). These findings are consistent with our findings which also show improvement in liver transaminases levels and normalization of ultrasonographic evidence in patients with NAFLD treated with PUFA.

In our study, HOMA-IR values, systolic blood pressure, fasting insulin, liver transaminases, HDL-C and triglycerides levels were significantly improved in patients receiving a diet plus PUFA after a 12-month treatment period. The same results were not noted in the placebo group. Others have also reported evidence suggesting that n-3 PUFAs are able to reduce blood pressure (32) and that they have favorable effects on plasma lipids levels (33). Spadaro et al (34) also reported that serum ALT and lipid levels are decreased after n-3 PUFA treatment. The results of our study support these findings. Zhu et al (35) performed a randomized clinical trial with a large sample size. In 144 patients with NAFLD and mixed hyperlipidemia, the efficacy of n-3 PUFA from seal oils was evaluated. At the end of the treatment period (24 wk), total symptom scores, ALT, triglycerides levels and fatty liver scores decreased more significantly in the group treated with 2000 mg of seal oils than in the placebo group. Finally, in another study (36) in which the diagnosis of NAFLD was confirmed by biopsy, 60 children were randomly assigned to receive DHA 250 mg/day, DHA 500 mg/day or placebo. The duration of the treatment was 6 months. DHA supplementation was associated with amelioration of severe steatosis compared to a placebo after 6 months. At the end of our study, as compared to the pretreatment values, the grade of the steatosis and ALT/AST levels showed significant decreases at months 3, 6, 9 and 12 in both PUFA and control groups. However, we found significant improvements in the PUFA group when compared to the placebo group.

The gold standard for diagnosis of NAFLD is liver biopsy, but it is not frequently performed in NAFLD patients due to its low acceptance rate (37). In the present study, ultrasonography was performed to detect and monitor the changes in the liver. Lack of histological findings is a major drawback of this investigation.

In conclusion, the results of this randomized study of twelve months’ duration have demonstrated the positive effects of diet plus n-3 PUFA in NAFLD patients as compared with those receiving diet plus placebo. The n-3 PUFA treated patients showed a reduction in fasting insulin, triglyceride, ALT, AST levels and HOMA-IR values. In addition, significant benefits were noted in the hepatic ultrasonographic pattern following this treatment.

### Statement of Authorship

MB and NH have carried out the literature review, selection of sample size, data analysis, study design and writing the manuscript of this study. OP and BD helped with the design, data analysis and drafting of the manuscript. All other authors also participated in the study design, conduction of the study and finalizing the manuscript.

## Figures and Tables

**Table 1:**
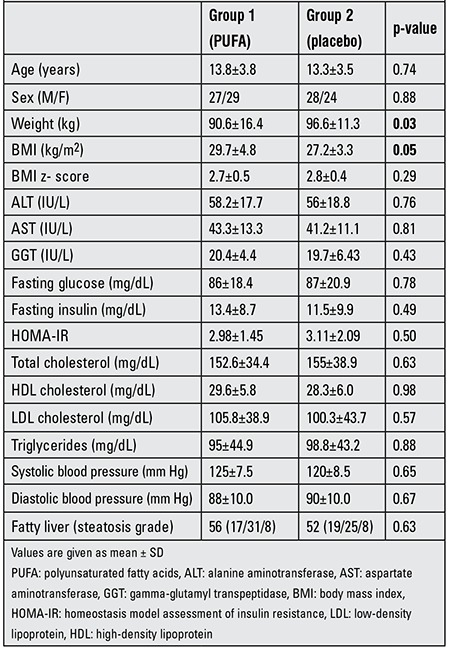
Characteristics of the two groups (group 1 receiving PUFA, group 2 - placebo) at baseline.

**Table 2:**
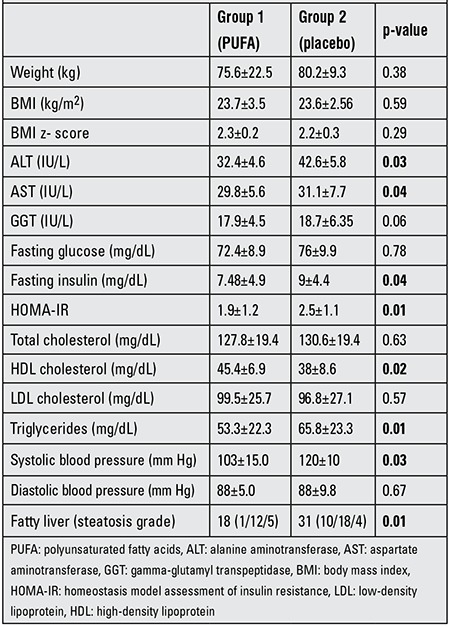
Characteristics of the two groups (group 1 receiving PUFA, group 2 - placebo) after 12 months.
